# Distributed Compressed Sensing Based Ground Moving Target Indication for Dual-Channel SAR System

**DOI:** 10.3390/s18072377

**Published:** 2018-07-21

**Authors:** Jing Liu, Xiaoqing Tian, Jiayuan Jiang, Kaiyu Huang

**Affiliations:** Ministry of Education Key Laboratory of Intelligent and Network Security, School of Electronics and Information Engineering, Xi’an Jiaotong University, Xi’an 710049, Shaanxi, China; tianxiaoqing2017@stu.xjtu.edu.cn (X.T.); jyjiang@stu.xjtu.edu.cn (J.J.); huangkaiyu@stu.xjtu.edu.cn (K.H.)

**Keywords:** distributed compressed sensing, Variational Bayesian, dual-channel SAR, ground moving-target indication

## Abstract

The dual-channel synthetic aperture radar (SAR) system is widely applied in the field of ground moving-target indication (GMTI). With the increase of the imaging resolution, the resulting substantial raw data samples increase the transmission and storage burden. We tackle the problem by adopting the joint sparsity model 1 (JSM-1) in distributed compressed sensing (DCS) to exploit the correlation between the two channels of the dual-channel SAR system. We propose a novel algorithm, namely the hierarchical variational Bayesian based distributed compressed sensing (HVB-DCS) algorithm for the JSM-1 model, which decouples the common component from the innovation components by applying variational Bayesian approximation. Using the proposed HVB-DCS algorithm in the dual-channel SAR based GMTI (SAR-GMTI) system, we can jointly reconstruct the dual-channel signals, and simultaneously detect the moving targets and stationary clutter, which enables sampling at a further lower rate in azimuth as well as improves the reconstruction accuracy. The simulation and experimental results show that the proposed HVB-DCS algorithm is capable of detecting multiple moving targets while suppressing the clutter at a much lower data rate in azimuth compared with the compressed sensing (CS) and range-Doppler (RD) algorithms.

## 1. Introduction

Synthetic aperture radar (SAR) is a kind of high resolution imaging radar, which is capable of working at long distances, all-weather, and day and night. In the SAR system, the scene is observed by an antenna at different positions in the azimuth direction. The coherent information recorded at the different positions is used to synthesize a very long antenna to improve the azimuth resolution. The dual-channel SAR system is widely applied in the field of ground moving-target indication (GMTI). In practice, the Doppler shift of the stationary clutter spreads the Doppler bandwidth of the clutter returns due to the motion of the platform. Accordingly, the Doppler bandwidth of the clutter will mask that of moving targets [1]. Dual-channel SAR can effectively suppress the clutter, which is propitious to slowly moving targets detection [2]. Some traditional approaches, such as the displaced phase center antenna (DPCA) approach, and along-track interferometry (ATI) detection [3], have achieved good results. However, relying on the Nyquist sampling theorem, dual-channel SAR is obliged to transmit and store substantial raw data samples, which limits its application in practice.

Compressed sensing (CS) [4,5,6,7], as a novel sparse reconstruction technique, is able to recover the signals that have been sampled below the traditional Nyquist sampling rate. It provides a good solution for the transmission and storage of substantial raw data samples. CS aims at minimizing the number of measurements and attempts to recover the original signal from these necessary measurements by solving an $\ell_{0}$-norm optimization problem, or equivalently a convex $\ell_{1}$-norm optimization problem under the restricted isometry property (RIP) condition [8].

A lot of valuable literature on CS-based SAR imaging and the GMTI system has been published in recent years. A CS-based SAR imaging algorithm is proposed in [9], which demonstrates that CS has the potential to eliminate the need for the pulse compression matched filter at the receiver, and reduce the required receiver analog-to-digital conversion bandwidth. In [10], CS is applied to randomly select lines in azimuth from the signals after range compression, which allows the implementation of wide-swath modes without reducing the azimuth resolution. Sun et al. [11] propose a CS-based method for joint sparse recovery of all channel or sub-aperture images. In [12], a fast CS-based SAR imaging algorithm is proposed to save the computational cost both in time and memory. In [13], a phase error correction method is proposed for the CS-based radar imaging system based on approximated observation, which improves the defocus caused by the phase error. In [14], CS is utilized in tomographic SAR system, extended to the SAR elevation direction for 3-D imaging. In the field of GMTI, in [15] CS is used to estimate the velocities and positions of moving targets. In [16,17], a CS-based space-time adaptive processing (STAP) algorithm is proposed to detect the moving targets. The above CS-based algorithms can efficiently reduce data rate and improve image quality, as well as improve anti-jamming performance. However, for the dual-channel SAR based GMTI (SAR-GMTI) system, with the increase of the imaging resolution, the resulting substantial raw data samples aggravate transmission and storage load. Thus it is required to develop an efficient algorithm that can further reduce the data rate.

In this work, we tackle the problem by exploiting the correlation between two channels of the dual-channel SAR system. The antennas of dual-channel SAR are configured in the along-track direction. Thus the SAR images acquired by each channel are highly correlated during a flight along the track. Using distributed compressed sensing (DCS) based algorithms, we can jointly reconstruct the dual-channel signals, which enables sampling at a further lower rate in azimuth as well as improves the reconstruction accuracy.

DCS [18] exploits both intra-signal and inter-signal correlations. Three different joint sparsity models have been proposed, i.e., joint sparsity model 1 (JSM-1), joint sparsity model 2 (JSM-2) and joint sparsity model 3 (JSM-3). In JSM-1, a sparse signal consists of a common component and an innovation component. Such signals may exist when large-scale phenomena affects all signal sources and local phenomena affects specific individual source. In JSM-2, all signals are constructed from the same sparse set of basis vectors, but with different coefficient values. JSM-3 extends JSM-1 so that the common component needs no longer to be sparse in any basis. In this work, the echo signal of dual-channel SAR is the combination of the signals from both stationary clutter and moving targets. After time delay and phase compensation, the signal from stationary clutter is common to all channels, while the signals from moving targets are specific and different across different channels. Thus the JSM-1 model is adopted, considering that the signal from stationary clutter can be treated as the common component, while those from moving targets can be treated as the innovation components.

Many reconstruction algorithms have been proposed for the JSM-1 model, either in a central manner or a distributed manner. The central recovery methods assume the presence of a fusion center where all information is gathered. The joint recovery strategy (JRS) [18,19] is to stack the common component and all innovation components together as one single sparse signal, and reconstruct the single sparse signal via $\ell_{0}$-norm minimization or $\ell_{1}$-norm minimization. There are well developed algorithms such as subspace pursuit (SP), basic pursuit (BP), iterative hard thresholding (IHT), etc., to solve the minimization problem. However, JRS is computationally expensive since the dimension of the sparse signal is multiplied by stacking. As another branch of the methods, the distributed recovery performs the reconstruction in the network, with no fusion center. The Texas Hold ’Em algorithm [20] separates the estimation of the common component and innovation components by the measurement decomposition process. Valsesia et al. [21] exploit the side information from one of the signals to reconstruct the innovation components. Recently Sundman et al. [22] propose the parallel pursuit with side information (SIPP) algorithm, which extends SP by using the side information of common support set at each iteration. SIPP can reconstruct the signals in a distributed manner, thus achieving much less computing time than JRS. This implies that the information of support set can be utilized to accelerate the reconstruction process. In [23], an alternating direction method of multipliers (ADMM) based algorithm is proposed for the JSM-1 model in a decentralized framework, which exploits the local cooperation between nodes.

In this work, we consider dealing with the JSM-1 model in a different way, i.e., in a Bayesian framework. Bayesian methods essentially approximate the posterior distributions of sparse signals according to the prior knowledge and data, which is effective in dealing with uncertain models and large noises. We consider using the variational Bayesian algorithm for the JSM-1 model. Variational Bayesian (VB) inference [24] obtains the optimal solution by iterating over a set of mutually dependent equations, providing a good approximation to the exact posterior with a readily monitored convergence.

Variational Bayesian has been widely applied in sparse signal reconstruction [25,26]. In this work, we propose a novel algorithm, namely the hierarchical variational Bayesian based distributed compressed sensing (HVB-DCS) algorithm for the JSM-1 model, which decouples the common component from the innovation components by applying variational approximation. The proposed algorithm is capable of approximating the complex signal in a hierarchical framework. Moreover, we apply the proposed HVB-DCS algorithm for signal reconstruction in the dual-channel SAR based GMTI system. Based on the received signal from both the moving targets and the stationary clutter, we decouple the common component (stationary clutter) from the innovation components (moving targets). We derive separable PDF functions for common component and innovation components, respectively. We can then obtain the reconstructed signal (including the common and innovation components) by applying the maximum a posteriori (MAP) estimation. The performance of the algorithm is verified by both simulation on point targets, and experiment on real SAR data.

The main contribution of this paper is threefold. First, we model the received signals of the dual-channel SAR-GMTI system in the JSM-1 model of DCS, by exploiting the correlation between the two channels. Secondly, we propose a novel HVB-DCS algorithm for the JSM-1 model, which decouples the common component from the innovation components by applying variational approximation. The proposed HVB-DCS algorithm approximates the complex signal in a hierarchical framework. Thirdly, we apply the proposed HVB-DCS algorithm for signal reconstruction in the dual-channel SAR-GMTI system. In the framework of the JSM-1 model, the proposed algorithm can simultaneously generate the stationary clutter (common component) and the moving targets (innovation components), which omits the DPCA process adopted in the traditional GMTI system. The simulation and experimental results show that the proposed HVB-DCS algorithm is capable of detecting multiple moving targets while suppressing the clutter at a much lower data rate compared with the CS and range-Doppler (RD) algorithms.

The organization of this paper is as follows. First, we briefly introduce the theory of DCS in Section 2. We then present the signal model of dual-channel SAR system in Section 3. The DCS-based dual-channel SAR-GMTI system is introduced in Section 4, where the echo signals of dual-channel SAR-GMTI system are represented in the JSM-1 model. The HVB-DCS algorithm is proposed in Section 5, which decouples the common component from the innovation components by applying variational approximation, in a hierarchical framework. The simulation and experimental results are shown in Section 6, and the conclusions are summarized in Section 7.

## 2. Brief Introduction to Distributed Compressed Sensing

CS [4] is an emerging field for signal and image processing. CS aims to recover a sparse signal with a reduced number of linear measurements, which is significantly less than that required by the traditional bandwidth constraint based on the Shannon-Nyquist sampling theorem. CS theory has been widely applied in various fields, such as magnetic resonance imaging [27], target tracking [28], biomedical monitoring [29], computer-generated holography [30], machine learning [31], signal acquisition in wireless sensor networks [32], image/video compression [33], etc.

As an important branch of CS, DCS [18] considers a scenario where correlated sparse signals are captured from multiple sources. DCS aims to exploit the inter-signal and intra-signal correlations of those sparse signals, so that the ensemble of signals can be reconstructed from fewer measurements than the standard CS approach requires. Baron et al. in [18] propose three various joint sparsity models (JSMs) for the correlated signals, namely JSM-1, JSM-2 and JSM-3. Among them, JSM-1 is capable of modeling a wide range of scenarios where large-scale phenomena affect all signal sources and local phenomena affect specific individual source. In this work, we focus on the JSM-1 model.

### 2.1. Joint Sparsity Model-1

In the JSM-1 framework, an ensemble of vectors (signals) ${\left\{x_{j}\right\}}_{j=1}^{J}$ are jointly sparse if each vector is sparse and comprised of a common component and an innovation component, as

(1) $$x_{j}=z_{c}+z_{j},\;j\in \left\{1,2,\cdots ,J\right\},$$

where $z_{c}\in {ℜ}^{N}$ denotes the common component of sparse vector $x_{j}$, and $z_{j}\in {ℜ}^{N}$ denotes the innovation component of $x_{j}$. Both $z_{c}$ and $z_{j}$ are supposed to be sparse.

The DCS problem consists in reconstructing jointly sparse vectors from their measurements, which are obtained by

(2) $$y_{j}=A_{j}x_{j},$$

where $A_{j}\in {ℜ}^{M_{j}\times N}$ is the measurement matrix, $y_{j}\in {ℜ}^{M_{j}}$ is the measurement vector, and $M_{j}$ is the number of measurements.

### 2.2. The Joint Recovery Strategy for the JSM-1 Model

JRS is the first proposed algorithm for the JSM-1 model. The main idea is to stack the common component $z_{c}$ and all innovation components ${\left\{z_{j}\right\}}_{j=1}^{J}$ together to form one sparse vector $\tilde{z}$ with multiple dimension (J+1)N, and the similar process for measurement vectors ${\left\{y_{j}\right\}}_{j=1}^{J}$ and measurement matrices ${\left\{A_{j}\right\}}_{j=1}^{J}$, as

(3) $$\begin{array}{c} \tilde{z}:=\left[\begin{array}{c} z_{c}\\ z_{1}\\ z_{2}\\ \vdots \\ z_{J} \end{array}\right],\tilde{y}:=\left[\begin{array}{c} y_{1}\\ y_{2}\\ \vdots \\ y_{J} \end{array}\right],\tilde{A}:=\left[\begin{array}{ccccc} A_{1} & A_{1} & 0 & \cdots & 0\\ A_{2} & 0 & A_{2} & \cdots & 0\\ \vdots & \vdots & \vdots & \ddots & \vdots \\ A_{J} & 0 & 0 & \cdots & A_{J} \end{array}\right]. \end{array}$$

The *J* measurement Equations (2) can be formulated in one compact form as

(4) $$\tilde{y}=\tilde{A}\tilde{z},$$

resulting in the formulation of a standard CS problem. Then the sparse vector $\tilde{z}$ is reconstructed based on Equation (4) via well developed algorithms of CS, such as orthogonal matching pursuit (OMP) and BP. Finally, the jointly sparse vectors ${\left\{x_{j}\right\}}_{j=1}^{J}$ are reconstructed by adding the reconstructed common component to each reconstructed innovation component.

However, the computational costs of JRS for the JSM-1 model increase dramatically when the dimension of sparse vector increases. So it is necessary to develop another more efficient recovery strategy for the JSM-1 model.

## 3. Signal Model of the Dual-Channel SAR System

**Figure 1:**
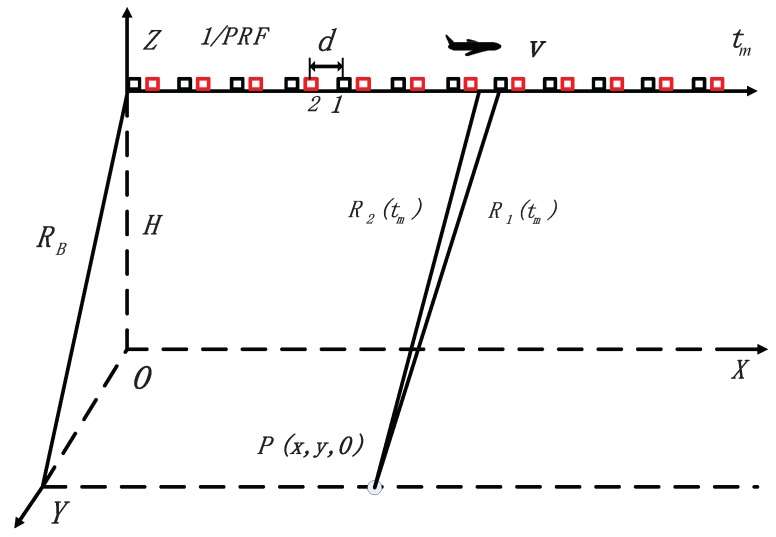
Geometry of the dual-channel SAR system.

This section introduces the signal model of a dual-channel SAR system, which is the same as that in [34]. The readers can refer to [34] for more details about the signal model. The geometry relationship between the flying platform and moving target is shown in Figure 1, where *v* and *H* denote velocity and height of the platform respectively. Channel one periodically transmits and receives pulses, while channel two receives pulses. The distance between the two channels is *d*. $t_{m}$ is the slow time in azimuth. PRF represents the pulse repetition frequency. The target is denoted by *P*. $R_{B}$ denotes the nearest slant range between the platform and target, whereas $R_{1}\left(t_{m}\right)$ is the instantaneous slant range between channel one and the moving target, and the similar definition for $R_{2}\left(t_{m}\right)$.

**Figure 2:**
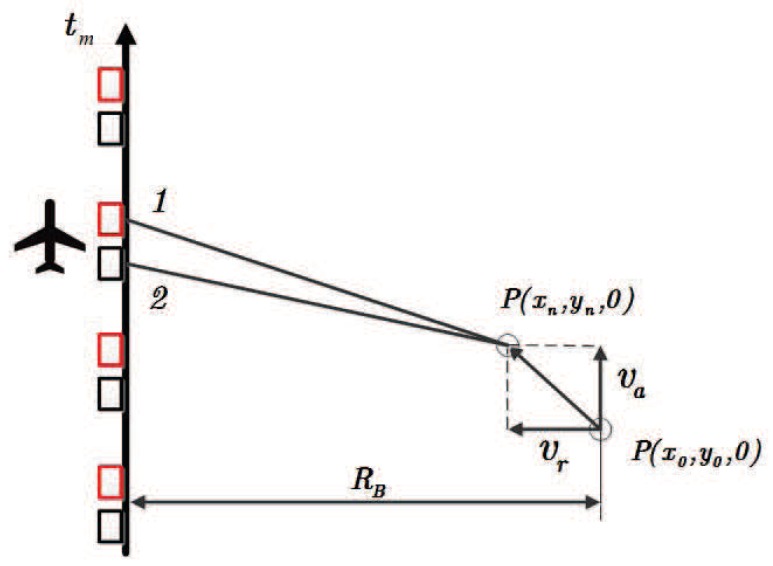
Slant range history geometry of the moving target.

In order to facilitate the analysis, the slant range history geometry of the moving target is shown in Figure 2. $v_{a}$ and $v_{r}$ denote the along-track and cross-track velocities of the moving target on the slant range plane, respectively. The instantaneous slant ranges $R_{1}\left(t_{m}\right)$ and $R_{2}\left(t_{m}\right)$ are respectively defined as

(5) $$\begin{array}{c} R_{1}\left(t_{m};R_{B}\right)=\sqrt{{\left(vt_{m}-v_{a}t_{m}\right)}^{2}+{\left(R_{B}-v_{r}t_{m}\right)}^{2}}\approx R_{B}-v_{r}t_{m}+\frac{{\left(v-v_{a}\right)}^{2}}{2R_{B}}t_{m}^{2}, \end{array}$$

and

(6) $$\begin{array}{c} R_{2}\left(t_{m};R_{B}\right)=\sqrt{{\left(vt_{m}-v_{a}t_{m}-d\right)}^{2}+{\left(R_{B}-v_{r}t_{m}\right)}^{2}}\approx R_{B}-v_{r}t_{m}+\frac{{\left[\left(v-v_{a}\right)t_{m}-d\right]}^{2}}{2R_{B}}, \end{array}$$

where

$$\begin{array}{c} t_{m}\in \left\{t_{1},t_{2}\cdots ,t_{i},\cdots ,t_{M}\right\}-\frac{t_{1}+t_{M}}{2}, \end{array}$$

and *M* denotes the number of pulses transmitted by antennas.

For channel one, the received signal of moving target after demodulation and range compression can be expressed as

(7) $$\begin{array}{c} s_{1}\left(t,t_{m}\right)=\sigma G\mathrm{sinc}\left(\Delta B\left(t-\frac{2R_{1}\left(t_{m};R_{B}\right)}{c}\right)\right)\omega_{a}\left(t_{m}\right)\exp\left(-j\frac{4\pi }{\lambda }R_{1}\left(t_{m};R_{B}\right)\right). \end{array}$$

The received signal for channel two is similarly defined as

(8) $$\begin{array}{c} s_{2}\left(t,t_{m}\right)=\sigma G\mathrm{sinc}\left(\Delta B\left(t-\frac{R_{1}\left(t_{m};R_{B}\right)+R_{2}\left(t_{m};R_{B}\right)}{c}\right)\right)\omega_{a}\left(t_{m}\right)\exp(-j\frac{2\pi }{\lambda }\left(R_{1}\left(t_{m};R_{B}\right)+R_{2}\left(t_{m};R_{B}\right)\right)). \end{array}$$

In the above expressions, *t* is the fast time in range, σ denotes the complex reflectivity of the target, and *G* is the range compression gain. ΔB is the bandwidth of transmitted signal. sinc(·) is the envelope after range compression. $\omega_{a}\left(\cdot \right)$ is the azimuth windowing function. *c* and λ represent the speed of light and the wavelength of transmitted signal, respectively.

## 4. Distributed Compressed Sensing Based Dual-Channel SAR-GMTI System

### 4.1. Sparse Representation for Individual Channel

**Figure 3:**
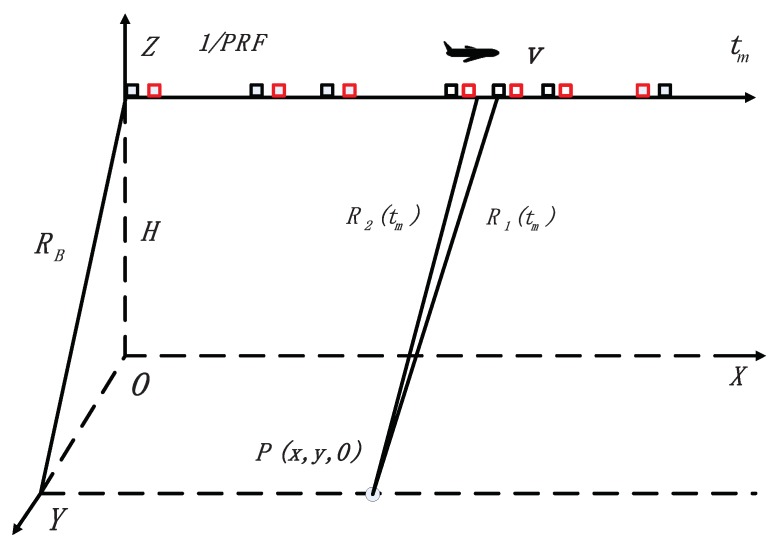
Random sampling mode in the azimuth direction.

In this section, the random sampling mode in the azimuth direction [34] is adopted for the dual-channel SAR system, which is shown in Figure 3. In this mode, the pulses in azimuth are randomly transmitted and received, which results in time gaps within a coherent processing interval (CPI) where no echoes are recorded. Moreover, we assume that the sensors remain still between transmission and reception of a pulse.

For channel one, we can obtain Equation (9) by substituting Equation (5) into Equation (7).

(9) $$\begin{array}{c} s_{1}\left(t,t_{m}\right)=\sigma G\mathrm{sinc}\left(\Delta B\left(t-\frac{2R_{B}}{c}\right)\right)\omega_{a}\left(t_{m}\right)\exp\left(-j\frac{4\pi }{\lambda }R_{B}\right)\exp\left(j2\pi f_{dc}t_{m}+j\pi \gamma_{m}\left(R_{B}\right)t_{m}^{2}\right), \end{array}$$

where $f_{dc}=2v_{r}/\lambda$ is the Doppler centroid frequency and $\gamma_{m}\left(R_{B}\right)=-2{\left(v-v_{a}\right)}^{2}/\left(\lambda R_{B}\right)$ is the corresponding Doppler chirp rate. Thus Equation (9) can be expanded as

(10) $$\begin{array}{c} s_{1}\left(t,t_{m}\right)=\sigma G\mathrm{sinc}\left(\Delta B\left(t-\frac{2R_{B}}{c}\right)\right)\omega_{a}\left(t_{m}\right)\exp\left(-j\frac{4\pi }{\lambda }R_{B}\right)\exp\left(j2\pi \frac{v_{r}^{2}R_{B}}{\lambda {\left(v-v_{a}\right)}^{2}}\right)\\ \;\;\;\;\;\;\;\;\times \exp\left(-j2\pi \frac{{\left(v-v_{a}\right)}^{2}}{\lambda R_{B}}{\left(t_{m}-\frac{v_{r}R_{B}}{{\left(v-v_{a}\right)}^{2}}\right)}^{2}\right). \end{array}$$

Assuming that the cross-track velocity of the moving point target is relatively slow, the range migration through resolution cells does not take place.

*Remark*: If the cross-track velocity of the moving target is high enough, the Doppler centroid frequency $f_{dc}$ may exceed the limit of PRF, which induces the Doppler ambiguity problem and an additional range walk [35]. For the condition of high cross-track velocity, an additional range walk correction step is required after range compression. As the cross-track velocity is unknown, it is required to search the Doppler ambiguity number and estimate the cross-track velocity. In recent years, many algorithms have been proposed to estimate the Doppler ambiguity number and the cross-track velocity, e.g., the road slope-aided algorithm [36] and the range walk correcting-based algorithm [37]. In the future work, we will combine the proposed HVB-DCS algorithm and the above-mentioned estimation algorithms to tackle the high cross-track velocity problem.

The received signal at a given range bin data, after demodulation and range compression, can be rewritten as

(11) $$\begin{array}{c} \begin{array}{c} s_{1}\left(t_{m}\right)=\rho^{\left(1\right)}\omega_{a}\left(t_{m}\right)\exp\left(-j2\pi \frac{{\left(v-v_{a}\right)}^{2}}{\lambda R_{B}}{\left(t_{m}-\frac{v_{r}R_{B}}{{\left(v-v_{a}\right)}^{2}}\right)}^{2}\right), \end{array} \end{array}$$

where

(12) $$\begin{array}{c} \begin{array}{c} \rho^{\left(1\right)}=\sigma G\mathrm{sinc}\left(\Delta B\left(t-\frac{2R_{B}}{c}\right)\right)\exp\left(-j\frac{4\pi }{\lambda }R_{B}\right)\exp\left(j2\pi \frac{v_{r}^{2}R_{B}}{\lambda {\left(v-v_{a}\right)}^{2}}\right). \end{array} \end{array}$$

We assume that there are no more than $N_{0}$ scattering centers which can be distinguished in the synthetic aperture time $T_{0}$. The coordinates of these scatterers in the azimuth direction are $x_{n}\left(n=1,2,\cdots ,N_{0}\right)$. The received signal at a given range bin data, after demodulation and range compression, can then be represented as a combination of the echo signals from $N_{0}$ scattering centres as,

(13) $$\begin{array}{c} \begin{array}{c} s_{1}\left(t_{m}\right)=\sum_{n=1}^{N_{0}}\rho_{n}^{\left(1\right)}\omega_{a}\left(t_{m}-x_{n}/v\right)\exp\left(-j2\pi \frac{{\left(v-v_{a}\right)}^{2}}{\lambda R_{B}}{\left(t_{m}-x_{n}/v-\frac{v_{r}R_{B}}{{\left(v-v_{a}\right)}^{2}}\right)}^{2}\right), \end{array} \end{array}$$

where

(14) $$\rho_{n}^{\left(1\right)}=\sigma_{n}G\mathrm{sinc}\left(\Delta B\left(t-\frac{2R_{B}}{c}\right)\right)\exp\left(-j\frac{4\pi }{\lambda }R_{B}\right).$$

The along-track velocity of the target defocuses the image, thus in order to facilitate the analysis, we only consider the cross-track velocity and let $v_{a}=0$.

Next, we represent the signal received by channel one in a standard CS framework. We first build the measurement matrix according to Equation (13), as

(15) $$\begin{array}{c} \Phi_{1}={\left[s_{0}\left(-\frac{N}{2}+1\right),s_{0}\left(-\frac{N}{2}\right),\cdots ,s_{0}\left(0\right),\cdots ,s_{0}\left(\frac{N}{2}-1\right),s_{0}\left(\frac{N}{2}\right)\right]}_{M\times N}, \end{array}$$

where,

(16) $$s_{0}\left(i\right)={\left[s_{0}\left(t_{1}-i\Delta \tau \right),s_{0}\left(t_{2}-i\Delta \tau \right),\cdots ,s_{0}\left(t_{M}-i\Delta \tau \right)\right]}_{M}^{T},$$

(17) $$s_{0}\left(t_{m}-i\Delta \tau \right)=\left\{\begin{array}{ccc} \exp\left(j\pi \gamma_{m}\left(R_{B}\right){\left(t_{m}-i\Delta \tau \right)}^{2}\right)\; & , & \left|t_{m}-i\Delta \tau \right|\leq T/2,\\ 0\;\;\;\;\;\;\;\;\;\;\;\; & , & \left|t_{m}-i\Delta \tau \right|>T/2, \end{array}\right.$$

$$\begin{array}{c} i\in \left\{-\frac{N}{2}+1,\cdots ,0,\cdots ,\frac{N}{2}\right\}, \end{array}$$

$\gamma_{m}\left(R_{B}\right)=-2{\left(v-v_{a}\right)}^{2}/\left(\lambda R_{B}\right)$, *T* is the full synthetic aperture time, Δτ=1/PRF, and $N\geq (T_{0}+T)/\Delta \tau$. *N* is the number of samples in the azimuth direction. *M* is far less than *N*. The measurement matrix captures the contribution to the received signal of a point target.

We then define the measurement vector $s_{1}\in C^{M}$ as the received signal for a given range bin after range compression by channel one, $s_{1}={\left[s_{1}\left(t_{1}\right),s_{1}\left(t_{2}\right),\cdots ,s_{1}\left(t_{M}\right)\right]}^{T}$, and the sparse vector $\rho_{1}\in C^{N}$ as the complex image for a given range bin, $\rho_{1}={\left[\rho_{1}^{\left(1\right)},\rho_{2}^{\left(1\right)},\cdots ,\rho_{N}^{\left(1\right)}\right]}^{T}$. Thus we can obtain the standard equation in CS for channel one as

(18) $$\begin{array}{c} s_{1}=\Phi_{1}\rho_{1}. \end{array}$$

Similarly, for channel two, Equation (6) is substituted into Equation (8), resulting in

(19) $$\begin{array}{cc} s_{2}\left(t,t_{m}\right) & =\sigma G\mathrm{sinc}\left(\Delta B\left(t-\frac{2R_{B}}{c}\right)\right)\omega_{a}\left(t_{m}\right)\exp\left(-j\frac{4\pi }{\lambda }R_{B}\right)\exp\left(j2\pi \frac{v_{r}^{2}R_{B}}{\lambda {\left(v-v_{a}\right)}^{2}}\right)\exp\left(-j\pi \frac{d^{2}}{2\lambda R_{B}}\right)\\ & \times \exp\left(-j2\pi \frac{{\left(v-v_{a}\right)}^{2}}{\lambda R_{B}}{\left(t_{m}-\frac{v_{r}R_{B}}{{\left(v-v_{a}\right)}^{2}}-\frac{d}{2\left(v-v_{a}\right)}\right)}^{2}\right)\exp\left(j\frac{2\pi }{\lambda }\frac{v_{r}d}{\left(v-v_{a}\right)}\right). \end{array}$$

Comparing Equation (19) (for channel two) with Equation (10) (for channel one), we can find that there is a phase difference $\exp\left(-j\pi d^{2}/\left(2\lambda R_{B}\right)\right)$ and a time delay $d/\left(2\left(v-v_{a}\right)\right)$ between the two channels. Thus the measurement matrix for channel two can be represented as

(20) $$\begin{array}{c} \Phi_{2}={\left[{s_{0}}^{\prime }\left(-\frac{N}{2}+1\right),{s_{0}}^{\prime }\left(-\frac{N}{2}\right),\cdots ,{s_{0}}^{\prime }\left(0\right),\cdots ,{s_{0}}^{\prime }\left(\frac{N}{2}-1\right),{s_{0}}^{\prime }\left(\frac{N}{2}\right)\right]}_{M\times N}, \end{array}$$

where,

(21) $$s_{0}^{\prime }\left(i\right)={\left[s_{0}^{\prime }\left(t_{1}-i\Delta \tau \right),s_{0}^{\prime }\left(t_{2}-i\Delta \tau \right),\cdots ,s_{0}^{\prime }\left(t_{M}-i\Delta \tau \right)\right]}_{M}^{T},$$

(22) $${s_{0}}^{\prime }\left(t_{m}-i\Delta \tau \right)=\left\{\begin{array}{ccc} \exp\left(j\pi \gamma_{m}\left(R_{B}\right){\left(t_{m}-i\Delta \tau -\frac{d}{2\left(v-v_{a}\right)}\right)}^{2}\right)\; & , & \left|t_{m}-i\Delta \tau -\frac{d}{2(v-v_{a})}\right|\leq T/2,\\ 0\;\;\;\;\;\;\;\;\;\;\;\; & , & \left|t_{m}-i\Delta \tau -\frac{d}{2(v-v_{a})}\right|>T/2, \end{array}\right.$$

$$\begin{array}{c} i\in \left\{-\frac{N}{2}+1,\cdots ,0,\cdots ,\frac{N}{2}\right\}. \end{array}$$

We define the measurement vector $s_{2}\in C^{M}$ as the received signal for a given range bin after range compression by channel two, $s_{2}={\left[s_{2}\left(t_{1}\right),s_{2}\left(t_{2}\right),\cdots ,s_{2}\left(t_{M}\right)\right]}^{T}$ and the sparse vector $\rho_{2}\in C^{N}$ as $\rho_{2}={\left[\rho_{1}^{\left(2\right)},\rho_{2}^{\left(2\right)},\cdots ,\rho_{N}^{\left(2\right)}\right]}^{T}$. Thus, we obtain the standard equation in CS for channel two as

(23) $$\begin{array}{c} s_{2}=\Phi_{2}\rho_{2}. \end{array}$$

As we can see, the measurement matrices are built using the echo signal models of moving targets as in Equations (13) and (19). In practice, it is difficult to construct the measurement matrices since the along-track velocity $v_{a}$ and cross-track velocity $v_{r}$ of the moving target are unknown. Thus, in order to facilitate the analysis and construction of the measurement matrices, we set the values of $v_{a}$ and $v_{r}$ to zero. The effects of the nonzero cross-track velocity and nonzero along-track velocity on SAR imaging are analyzed in detail through simulations performed in Section 6.1.

### 4.2. JSM-1 Model

For the $i^{th}$ scattering center, its corresponding reflection coefficients in channel one and channel two can be respectively represented as

(24) $$\begin{array}{c} \rho_{i}^{\left(1\right)}=\sigma_{i}G\mathrm{sinc}\left(\Delta B\left(t-\frac{2R_{B}}{c}\right)\right)\exp\left(-j\frac{4\pi }{\lambda }R_{B}\right)\exp\left(j\frac{2\pi v_{r}^{2}R_{B}}{\lambda v^{2}}\right), \end{array}$$

and

(25) $$\begin{array}{c} \rho_{i}^{\left(2\right)}=\sigma_{i}G\mathrm{sinc}\left(\Delta B\left(t-\frac{2R_{B}}{c}\right)\right)\exp\left(-j\frac{4\pi }{\lambda }R_{B}\right)\exp\left(j\frac{2\pi v_{r}^{2}R_{B}}{\lambda v^{2}}\right)\\ \;\;\times \exp\left(-j\pi \frac{d^{2}}{2R_{B}\lambda }\right)\exp\left(j\frac{2\pi }{\lambda }\left(v_{r}\frac{d}{v}\right)\right). \end{array}$$

Equations (24) and (25) demonstrate the close connection between the two channels, i.e., except the items of a fixed phase difference $\exp\left(-j\pi d^{2}/\left(2\lambda R_{B}\right)\right)$, and a varying phase difference $\exp\left(j\frac{2\pi }{\lambda }\left(v_{r}\frac{d}{v}\right)\right)$ changing with cross-track velocity in Equation (25), the other items on the reflection coefficients of the two channels are the same.

First, we consider compensating for the fixed phase difference on the $i^{th}$ reflection coefficient of channel two, $\rho_{i}^{\left(2\right)}$.

(26) $$\begin{array}{cc} \rho_{i}^{{\left(2\right)}^{\prime }} & =\rho_{i}^{\left(2\right)}\exp\left(j\pi d^{2}/2\lambda R_{B}\right)=\sigma_{i}G\mathrm{sinc}\left(\Delta B\left(t-\frac{2R_{B}}{c}\right)\right)\\ & \cdot \exp\left(-j\frac{4\pi }{\lambda }R_{B}\right)\exp\left(j\frac{2\pi v_{r}^{2}R_{B}}{\lambda v^{2}}\right)\exp\left(j\frac{2\pi }{\lambda }\left(v_{r}\frac{d}{v}\right)\right),i=1,\cdots ,N. \end{array}$$

The compensating procedure in Equation (26) is repeated for *N* reflection coefficients of channel two, resulting in a new sparse vector, i.e., $\rho_{2}^{\prime }={\left[\rho_{1}^{{\left(2\right)}^{\prime }},\rho_{2}^{{\left(2\right)}^{\prime }},\cdots ,\rho_{N}^{{\left(2\right)}^{\prime }}\right]}^{T}$, namely the compensated reflection coefficient vector. Furthermore, we can obtain the relationships between the reflection coefficient vectors of channel two, respectively after and before the compensating procedure, together with the reflection coefficient vector of channel one, as

(27) $$\begin{array}{c} \left[\begin{array}{c} \rho_{1}\\ \rho_{2}^{\prime } \end{array}\right]=\left[\begin{array}{cc} I & 0\\ 0 & \exp\left(j\pi \frac{d^{2}}{2\lambda R_{B}}\right)I \end{array}\right]\left[\begin{array}{c} \rho_{1}\\ \rho_{2} \end{array}\right], \end{array}$$

where I denotes an identity matrix of appropriate size.

Secondly, we build the JSM-1 model based on the original reflection coefficient vector of channel one, $\rho_{1}$, and the compensated reflection coefficient vector of channel two, $\rho_{2}^{\prime }$. The nonzero elements of $\rho_{1}$ consist of the reflection coefficients from both the stationary clutter and moving targets, which are denoted as $\rho_{s}^{\left(1\right)}$ and $\rho_{m}^{\left(1\right)}$, respectively. Similarly, the nonzero elements of $\rho_{2}^{\prime }$ consist of the reflection coefficients from both the stationary clutter and moving targets, which are denoted as $\rho_{s}^{{\left(2\right)}^{\prime }}$ and $\rho_{m}^{{\left(2\right)}^{\prime }}$, respectively. On the one hand, the reflection coefficients of stationary clutter are common to both $\rho_{1}$ and $\rho_{2}^{\prime }$, since $v_{r}=0$ and we have $\rho_{s}^{\left(1\right)}=\rho_{s}^{{\left(2\right)}^{\prime }}$. On the other hand, the reflection coefficients of moving targets are different for channel one and channel two, since $v_{r}\neq 0$, thus $\rho_{m}^{\left(1\right)}\neq \rho_{m}^{{\left(2\right)}^{\prime }}$. Let $z_{c}$ denote the vector consisting of the reflection coefficients of stationary clutter, $z_{1}$ and $z_{2}$ denote the vectors consisting of the reflection coefficients of moving targets observed in channel one and channel two, respectively. Then we have

(28) $$\begin{array}{c} \left[\begin{array}{c} \rho_{1}\\ \rho_{2}^{\prime } \end{array}\right]=\left[\begin{array}{ccc} I & I & 0\\ I & 0 & I \end{array}\right]\left[\begin{array}{c} z_{c}\\ z_{1}\\ z_{2} \end{array}\right]. \end{array}$$

We define $y={\left[s_{1}^{T}\;s_{2}^{T}\right]}^{T}$, and can obtain

(29) $$\begin{array}{c} \begin{array}{cc} y & =\left[\begin{array}{cc} \Phi_{1} & 0\\ 0 & \Phi_{2} \end{array}\right]\left[\begin{array}{c} \rho_{1}\\ \rho_{2} \end{array}\right]=\left[\begin{array}{cc} \Phi_{1} & 0\\ 0 & \Phi_{2} \end{array}\right]\left[\begin{array}{cc} I & 0\\ 0 & \exp\left(-j\pi \frac{d^{2}}{2\lambda R_{B}}\right)I \end{array}\right]\left[\begin{array}{c} \rho_{1}\\ \rho_{2}^{\prime } \end{array}\right]\\ & =\left[\begin{array}{cc} \Phi_{1} & 0\\ 0 & \Phi_{2} \end{array}\right]\left[\begin{array}{cc} I & 0\\ 0 & \exp\left(-j\pi \frac{d^{2}}{2\lambda R_{B}}\right)I \end{array}\right]\left[\begin{array}{ccc} I & I & 0\\ I & 0 & I \end{array}\right]\left[\begin{array}{c} z_{c}\\ z_{1}\\ z_{2} \end{array}\right]\\ & =\left[\begin{array}{ccc} \Phi_{1} & \Phi_{1} & 0\\ \Phi_{2}\exp\left(-j\pi \frac{d^{2}}{2\lambda R_{B}}\right) & 0 & \Phi_{2}\exp\left(-j\pi \frac{d^{2}}{2\lambda R_{B}}\right) \end{array}\right]\left[\begin{array}{c} z_{c}\\ z_{1}\\ z_{2} \end{array}\right] \end{array}. \end{array}$$

In Equation (29), the first equality is based on Equations (18) and (23), and the second equality holds considering the inverse of Equation (27).

Equation (29) is a standard JSM-1 model. By jointly reconstructing the common and innovation components of the above, the moving targets $z_{1}$ and $z_{2}$ can be separated. However, the computational costs of some existing algorithms for solving the above model increase dramatically when the dimension of sparse vector increases. So we develop a more efficient recovery strategy for this JSM-1 model.

## 5. The Hierarchical Variational Bayesian Based DCS Algorithm

### 5.1. Proposed Algorithm

In Bayesian modeling, all unknowns are treated as stochastic quantities with assigned probability distributions. Considering the effects of noise, the standard JSM-1 model can be presented as

(30) $$y_{k}=A_{k}\left(z_{c}+z_{k}\right)+v_{k},$$

where $y_{k}\in {ℜ}^{M}$, $A_{k}\in {ℜ}^{M\times N}$ and $v_{k}\in {ℜ}^{M}$ denote the measurement vector, the measurement matrix and noise vector, of channel k, respectively; $z_{c},z_{k}\in {ℜ}^{N}$ denote the common component and the innovation component respectively.

By applying a suitable prior distribution to $z_{c}$ and $z_{k}$, the sparsity can be guaranteed. However, it is important to allow the flexibility to model local characteristics of the signal, as the simple stationary sparse prior distribution is unable to meet the demand. For this reason, we propose an HVB-DCS algorithm for the JSM-1 model.

We adopt zero-mean Gaussian prior distributions for the common component and innovation components, respectively, which are given as

(31) $$\begin{array}{c} p\left(z_{c}|\alpha_{c}\right)=\prod_{n=1}^{N}N\left(z_{c}^{n}|0,{\left(\alpha_{c}^{n}\right)}^{-1}\right), \end{array}$$

and

(32) $$\begin{array}{c} p\left(z_{k}|\alpha_{k}\right)=\prod_{n=1}^{N}N\left(z_{k}^{n}|0,{\left(\alpha_{k}^{n}\right)}^{-1}\right), \end{array}$$

where $z_{c}^{n}$, $z_{k}^{n}$, $\alpha_{c}^{n}$, and $\alpha_{k}^{n}$ are the $n^{th}$ element of $z_{c}$, $z_{k}$, $\alpha_{c}$, and $\alpha_{k}$, respectively. The precision parameters $\alpha_{c}$ and $\alpha_{k}$ are constrained by treating them as random variables, with Gamma prior distributions as

(33) $$\begin{array}{c} p\left(\alpha_{c};a,b\right)=\prod_{n=1}^{N}Gamma\left(\alpha_{c}^{n}|a,b\right), \end{array}$$

and

(34) $$\begin{array}{c} p\left(\alpha_{k};c_{k},d_{k}\right)=\prod_{n=1}^{N}Gamma\left(\alpha_{k}^{n}|c_{k},d_{k}\right). \end{array}$$

Furthermore, we assume that the $m^{th}$ element of noise vector, $v_{k}^{m}\left(m=1,\cdots ,M\right),$ obeys an independent and identically distributed (i.i.d) zero-mean Gaussian distribution with inverse variance β, i.e., $v_{k}^{m}∼N\left(v_{k}^{m}|0,\beta^{-1}\right)$. Thus we can obtain the posterior distribution of noise vector $v_{k}$ as the product of that of the individual element as,

(35) $$p\left(v_{k}|\beta \right)=\prod_{m=1}^{M}N(v_{k}^{m}|0,\beta^{-1}).$$

We further assume a Gamma distribution as prior for the noise inverse variance β

(36) $$\begin{array}{c} p(\beta ;e,f)=Gamma(\beta |e,f), \end{array}$$

where *e* and *f* represent the shape and scale parameters of the Gamma distribution, respectively.

**Figure 4:**
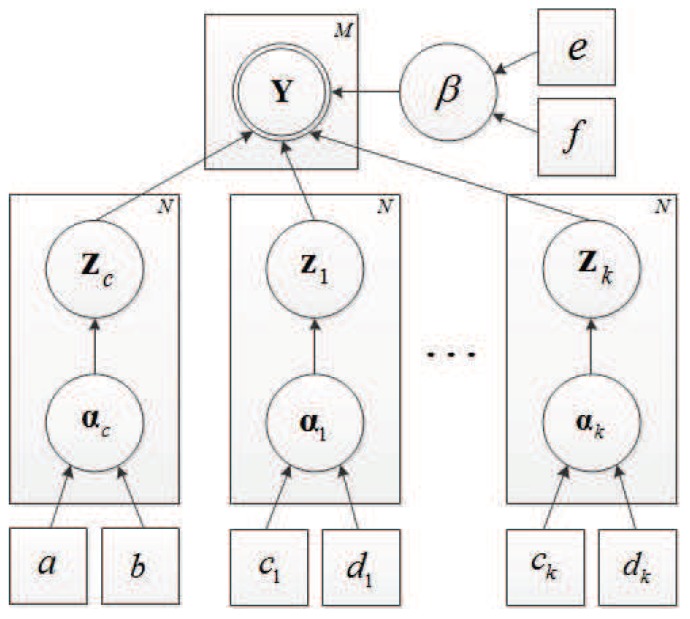
Graphical model for the JSM-1 model. Doubly circled node represents measurements, while single circled nodes represent hidden variables. Nodes denoted with squares correspond to hyperparameters. The directed edges represent the dependencies among the variables.

We define $Y=\{y_{1},y_{2},\ldots ,y_{k}\}$, $Z=\{z_{c},z_{1},\ldots ,z_{k},\alpha_{c},\ldots ,\alpha_{k},\beta \}$, and $\theta =\{a,b,c_{1}\ldots c_{k},d_{1}\ldots d_{k},e,f\}$ as the sets of measurement vectors, the hidden variables, and the hyperparameters of the imposed prior, respectively. Figure 4 describes the relationships among the measurements (indicated as a doubly circled node), the hidden variables (indicated as single circled nodes) and the hyperparameters (indicated as square nodes). The directed edges of the graphical model represent the dependencies among the variables. For instance, the measurements Y relies on the hidden variables $z_{c}$, $z_{1}$, $z_{2}$, ⋯, $z_{k}$ and β, while the hidden variable $z_{c}$ stochastically depends on the hidden variable $\alpha_{c}$, and $\alpha_{c}$ further relies on the model parameters a,b.

Next, we are to pursuit the posterior distribution of the hidden variable, p(Z|Y;θ). In [24], VB introduces a variational distribution q(Z) to approximate the true posterior distribution p(Z|Y;θ). First, the log marginal likelihood logp(Y;θ) can be represented as

(37) $$\begin{array}{c} \log p\left(Y;\theta \right)=F\left(q\left(Z\right),\theta \right)+KL\left(q\left(Z\right)\left|\right|p\left(Z|Y;\theta \right)\right), \end{array}$$

where $F\left(q\left(Z\right),\theta \right)$ is the free energy,

(38) $$\begin{array}{c} F\left(q\left(Z\right),\theta \right)=\int q\left(Z\right)log\left(\frac{p\left(Z,Y;\theta \right)}{q\left(Z\right)}\right)dZ, \end{array}$$

and $\mathrm{KL}\left(q\left(Z\right)\left|\right|p\left(Z|Y;\theta \right)\right)$ is the Kullback-Leibler (KL) divergence between the true posterior p(Z|Y;θ) and the variational distribution q(Z),

(39) $$\begin{array}{c} \mathrm{KL}\left(q(Z)||p(Z|Y;\theta )\right)=\int q\left(Z\right)\log\left(\frac{q(Z)}{p(Z|Y;\theta )}\right)dZ. \end{array}$$

The goal is to approximate the true posterior distribution by minimizing the KL. Due to the fact that $\mathrm{KL}\left(q(Z)||p(Z|Y;\theta )\right)\geq 0$, that objective can be achieved by maximizing $F\left(q(Z),\theta \right)$. For the JSM-1 model, we should not only find separable functions that approximate the posterior distribution of $z_{c}$ and $z_{k}$, but also make the integral $F\left(q(Z),\theta \right)$ tractable.

In order to meet the requirements, we can assume that q(Z) has a factorized form Equation (40). This factorized form stems from theoretical physics where it is called mean field theory [38].

(40) $$\begin{array}{c} q\left(Z\right)=q\left(z_{c}\right)q\left(z_{1}\right)\cdots q\left(z_{k}\right)q\left(\alpha_{c}\right)q\left(\alpha_{1}\right)\cdots q\left(\alpha_{k}\right)q\left(\beta \right). \end{array}$$

Optimizing the free energy in Equation (38) is realized by taking functional derivatives with respect to each of q(·) distributions while fixing the other distributions and setting ∂F(q)/∂q(·)=0. Furthermore, the computation of ∂F(q)/∂q(·)=0 (Assuming that $q\left(Z\right)=\prod_{i=1}^{K}q(z_{i})$) can be expressed as

(41) $$q\left(z_{j}\right)\propto \exp\left({\langle \ln p(Y,Z;\theta )\rangle }_{i\neq j}\right),$$

where ${\langle \cdot \rangle }_{i\neq j}$ denotes an expectation with respect to all factors except $q(z_{j})$.

Next, the approximate posterior distribution of each part in Equation (40) is calculated according to Equation (41). We first calculate the variational distribution for common component $z_{c}$, via Equation (42).

(42) $$\begin{array}{c} q\left(z_{c}\right)\propto \exp\left(\langle \ln p\left(Y,Z;\theta \right)\rangle_{q\left(Z\right)/q\left(z_{c}\right)}\right)\propto \exp\left(\sum_{k=1}^{K}\langle \ln p\left(y_{k}|z_{c},z_{k},\beta \right)\rangle_{q\left(z_{k}\right)q\left(\beta \right)}+\langle \ln p\left(z_{c}|\alpha_{c}\right)\rangle_{q\left(\alpha_{c}\right)}\right)\\ \;\;\;\propto N\left(z_{c};\mu_{c},\Sigma_{c}\right). \end{array}$$

Thus $z_{c}$ is confirmed to be Gaussian distributed, with covariance matrix $\Sigma_{c}$ and mean vector $\mu_{c}$, where

(43) $$\begin{array}{c} \Sigma_{c}={\left(\sum_{k=1}^{K}\langle \beta \rangle A_{k}^{T}A_{k}+\Lambda_{\langle \alpha_{c}\rangle }\right)}^{-1}, \end{array}$$

(44) $$\begin{array}{c} \mu_{c}=\langle \beta \rangle \Sigma_{c}\sum_{k=1}^{K}A_{k}^{T}\left(y_{k}-A_{k}\mu_{k}\right). \end{array}$$

In the above equations, $\Lambda_{\langle \alpha_{c}\rangle }\in {ℜ}^{N\times N}$ is a diagonal matrix with hyperparameters $\alpha_{c}^{n}$ (n=1,…,N), and *K* is the number of channels.

Secondly, we calculate the variational distributions for innovation components $z_{k},\;k=1,\cdots ,K$ via Equation (45).

(45) $$\begin{array}{cc} q\left(z_{k}\right) & \propto \exp\left({\langle \ln p\left(Y,Z;\theta \right)\rangle }_{q\left(Z\right)/q\left(z_{k}\right)}\right)\propto \exp\left({\langle \ln p\left(y_{k}|z_{c},z_{k},\beta \right)\rangle }_{q\left(z_{c}\right)q\left(\beta \right)}+{\langle \ln p\left(z_{k}|\alpha_{k}\right)\rangle }_{q\left(\alpha_{k}\right)}\right)\\ & \propto \exp\left(-\frac{\langle \beta \rangle }{2}{\langle {∥y_{k}-A_{k}\left(z_{c}+z_{k}\right)∥}_{2}^{2}\rangle }_{q\left(z_{c}\right)}-\frac{1}{2}z_{k}^{T}\Lambda_{\langle \alpha_{k}\rangle }z_{k}\right)\propto N\left(z_{k};\mu_{k},\Sigma_{k}\right). \end{array}$$

Thus, $z_{k}$ is also Gaussian distributed, with covariance matrix $\Sigma_{k}$ and mean vector $\mu_{k}$, where

(46) $$\begin{array}{c} \Sigma_{k}={\left(\langle \beta \rangle A_{k}^{T}A_{k}+\Lambda_{\langle \alpha_{k}\rangle }\right)}^{-1}, \end{array}$$

(47) $$\begin{array}{c} \mu_{k}=\langle \beta \rangle \Sigma_{k}A_{k}^{T}\left(y_{k}-A_{k}\mu_{c}\right). \end{array}$$

In the above equations, $\Lambda_{\langle \alpha_{k}\rangle }\in {ℜ}^{N\times N}$ is a diagonal matrix with hyperparameters $\alpha_{k}^{n}$ (k=1,…,K; n=1,…,N).

Thirdly, we calculate the variational distribution for the prior of common component.

(48) $$\begin{array}{cc} q\left(\alpha_{c}^{n}\right) & \propto \exp\left({\langle \ln p\left(z_{c}^{n}|\alpha_{c}^{n}\right)+\ln p\left(\alpha_{c}^{n};a,b\right)\rangle }_{q\left(z_{c}\right)q\left(\beta \right)}\right)\\ & \propto \exp\left(\frac{1}{2}\ln\alpha_{c}^{n}-\frac{1}{2}\langle {\left(z_{c}^{n}\right)}^{2}\rangle \alpha_{c}^{n}+\left(a-1\right)\ln\alpha_{c}^{n}-b\alpha_{c}^{n}\right)\\ & \propto \exp\left(\left(\frac{1}{2}+a-1\right)\ln\alpha_{c}^{n}-\left(b+\frac{1}{2}\langle {\left(z_{c}^{n}\right)}^{2}\rangle \right)\alpha_{c}^{n}\right)\propto Gamma\left(\alpha_{c}^{n};\tilde{a},{\tilde{b}}_{n}\right). \end{array}$$

Thus $\alpha_{c}^{n}$ is distributed as $Gamma(\alpha_{c}^{n};\tilde{a},{\tilde{b}}_{n})$, where

(49) $$\begin{array}{c} \tilde{a}=a+\frac{1}{2},{\tilde{b}}_{n}=b+\frac{1}{2}\langle {\left(z_{c}^{n}\right)}^{2}\rangle . \end{array}$$

Similarly, we can obtain the variational distributions for the prior of innovation components, as

(50) $$q\left(\alpha_{k}^{n}\right)\propto Gamma\left(\alpha_{k}^{n};{\tilde{c}}_{k},{\tilde{d}}_{k}^{n}\right),$$

where

(51) $$\begin{array}{c} {\tilde{c}}_{k}=c_{k}+\frac{1}{2},{\tilde{d}}_{k}^{n}=d_{k}+\frac{1}{2}\langle {\left(z_{k}^{n}\right)}^{2}\rangle . \end{array}$$

Finally, we calculate the approximate posterior distribution for the parameter of inverse variance β.

(52) $$\begin{array}{cc} q\left(\beta \right) & \propto \exp\left({\langle \ln p\left(Y,Z;\theta \right)\rangle }_{q\left(Z\right)/q\left(\beta \right)}\right)\propto \exp\left(\sum_{k=1}^{K}{\langle \ln p\left(y_{k}|z_{c},z_{k},\beta \right)\rangle }_{q\left(z_{c}\right)q\left(z_{k}\right)}+\ln p\left(\beta |e,f\right)\right)\\ & \propto \exp\left(\frac{KM}{2}\ln\beta -\frac{\beta }{2}\sum_{k=1}^{K}{\langle {∥y_{k}-A_{k}\left(z_{c}+z_{k}\right)∥}_{2}^{2}\rangle }_{q\left(z_{c}\right)q\left(z_{k}\right)}+\left(e-1\right)\ln\beta -f\beta \right)\\ & \propto Gamma\left(\beta ;\tilde{e},\tilde{f}\right). \end{array}$$

Thus β is distributed as $Gamma(\beta ;\tilde{e},\tilde{f})$, where

(53) $$\begin{array}{c} \tilde{e}=e+\frac{KM}{2},\\ \tilde{f}=f+\frac{1}{2}\left(\sum_{k=1}^{K}{\langle {∥y_{k}-A_{k}\left(z_{c}+z_{k}\right)∥}_{2}^{2}\rangle }_{q\left(z_{c}\right)q\left(z_{k}\right)}\right). \end{array}$$

The variational optimization proceeds by iteratively updating Equations (42), (45), (48), (50), and (52) until convergence occurs to hyperparameters θ. Finally, we can obtain the reconstructed signal by applying the maximum a posteriori estimation.

$$\begin{array}{c} {\hat{\rho }}_{k}=\underset{z_{c}+z_{k}}{\arg\max}p\left(Z|Y;\theta \right)=\underset{z_{c}}{\arg\max}q\left(z_{c}\right)+\underset{z_{k}}{\arg\max}q\left(z_{k}\right)=\mu_{c}+\mu_{k},\;k=1,2,\cdots ,K. \end{array}$$

The proposed HVB-DCS algorithm for solving the JSM-1 reconstruction problem is summarized in Algorithm 1.

**Algorithm 1: HVB-DCS** AlgorithmInput: A set of measurement vectors $\left\{y_{1},y_{2},\cdots ,y_{K}\right\}$ and corresponding measurement matrices$\{A_{1},A_{2},\cdots ,A_{K}\},k=1,\cdots ,K$.Output: The reconstructed signal ${\hat{\rho }}_{k}=\mu_{c}+\mu_{k}$, k=1,⋯,K.**Initialize the hyperparameters.** Set the initial values of the variables (*a*, *b*, $\left\{c_{k},k=1,\cdots ,K\right\}$,$\left\{d_{k},k=1,\cdots ,K\right\}$, *e*, *f*) as $10^{-6}$.
**Compute the variational distribution for the common component.**
Compute $\Sigma_{c}={\left(\sum_{k=1}^{K}\langle \beta \rangle A_{k}^{T}A_{k}+\Lambda_{\langle \alpha_{c}\rangle }\right)}^{-1}$, and $\mu_{c}=\langle \beta \rangle \Sigma_{c}\sum_{k=1}^{K}A_{k}^{T}\left(y_{k}-A_{k}\mu_{k}\right).$
**Compute the variational distribution for the prior of the common component.**
Update $q(\alpha_{c}^{n})$, compute
$\tilde{a}=a+\frac{1}{2},{\tilde{b}}_{n}=b+\frac{1}{2}\langle {\left(z_{c}^{n}\right)}^{2}\rangle .$

**Compute the variational distributions for the innovation components.**
Compute $\Sigma_{k}={\left(\langle \beta \rangle A_{k}^{T}A_{k}+\Lambda_{\langle \alpha_{k}\rangle }\right)}^{-1}$, and $\mu_{k}=\langle \beta \rangle \Sigma_{k}A_{k}^{T}\left(y_{k}-A_{k}\mu_{c}\right)$.
**Compute the variational distributions for the prior of the innovation components.**
Update $q\left(\alpha_{k}^{n}\right)$, compute
${\tilde{c}}_{k}=c_{k}+\frac{1}{2},{\tilde{d}}_{k}^{n}=d_{k}+\frac{1}{2}\langle {\left(z_{k}^{n}\right)}^{2}\rangle .$

**Compute the variational distribution for the prior of noise vector.**
Update q(β), compute
$\tilde{e}=e+\frac{KM}{2},$

$\tilde{f}=f+\frac{1}{2}\left(\sum_{k=1}^{K}{\langle {∥y_{k}-A_{k}\left(z_{c}+z_{k}\right)∥}_{2}^{2}\rangle }_{q\left(z_{c}\right)q\left(z_{k}\right)}\right).$

**Iterate steps 2 , 3 , 4 , 5 and 6 until convergence occurs to hyperparameters.**
**Output ${\hat{\rho }}_{k}=\mu_{c}+\mu_{k}$ for k=1,⋯,K**.

### 5.2. Complexity Analysis

We present the computational complexity of the proposed HVB-DCS algorithm, as well as the comparison to the CS-based joint sparsity recovery algorithm and the RD algorithm. The computational complexity of the HVB-DCS algorithm at each iteration is dominated by the inversion operations on an N×N matrix in Equations (43) and (46), and the multiplications of a matrix and a vector in Equations (44) and (47). The computational complexities of the matrix inversion and matrix-vector multiplication are $O\left(N^{3}\right)$ and $O\left(N^{2}\right)$, respectively. By using matrix inversion lemma [39], the complexity of the matrix inversion can be reduced to $O(M^{3})$. Thus the overall computational complexity of the HVB-DCS algorithm is $O\left(N_{t}\left(K+1\right)M^{3}\right)$, where $N_{t}$ is the total number of iterations. The small values of $N_{t}$ and *M* will result in acceptable computational complexity. In contrast, a large *N* will lead to high computational complexity, which makes the HVB-DCS algorithm impractical. To circumvent the high computational complexity problem, we can resort to a number of accelerating algorithms [40,41] to develop a more computationally efficient algorithm in the future work.

For the CS-based joint sparsity recovery algorithm, the dimensions of the sparse vector and the measurement vectors are 3N and 2M, respectively. Thus the computational complexity of the CS-based algorithm is $O(12NM^{2})$. For the RD algorithm, the computational complexity is dominated by the fast Fourier transform (FFT) and inverse fast Fourier transform (IFFT), which have computational complexities of $O(Mlog_{2}M)$ and $O(M/2log_{2}M)$, respectively. Thus, the overall computational complexity of the RD algorithm is $O\left(K\left(3N/2+1\right)Mlog_{2}M\right)$.

## 6. Simulations and Experiments

In this section, the performance of the proposed HVB-DCS algorithm is verified through both simulations and experiments. First, the effects of along-track and cross-track velocities on the SAR images reconstructed using the HVB-DCS algorithm are analyzed in Section 6.1. Secondly, the proposed HVB-DCS algorithm is compared with some classical GMTI algorithms, e.g., the RD [42] and CS [43] algorithms, based on point target simulation, in Section 6.2. Thirdly, the effects of problem size and data rate on complexity of the HVB-DCS algorithm are analyzed in Section 6.3. Finally, in Section 6.4, the proposed HVB-DCS algorithm is applied to the real background cluttered environment, where the real SAR data are collected by RADARSAT-1 satellite in the Vancouver region [42].

### 6.1. The Effects of Along-Track and Cross-Track Velocities on SAR Imaging

#### 6.1.1. The Effect of Cross-Track Velocity

First, we consider the influence of cross-track velocity on SAR imaging and take channel one as an example. The parameters of the simulated SAR radar system are listed in Table 1. Suppose that a moving target is at $\left(R_{B},x_{0}\right)$, with a given cross-track velocity $v_{r}$ and along-track velocity $v_{a}=0$ m/s. The received signal of the moving target by channel one, after demodulation and range compression, which is presented in Equation (13), is rewritten as

(54) $$\begin{array}{c} \begin{array}{c} s_{1}\left(t_{m}\right)=\rho_{0}^{\left(1\right)}\omega_{a}\left(t_{m}-k\Delta \tau \right)\exp\left(-j2\pi \frac{v^{2}}{\lambda R_{B}}{\left(t_{m}-k\Delta \tau -\frac{v_{r}R_{B}}{v^{2}}\right)}^{2}\right), \end{array} \end{array}$$

where $k\Delta \tau =x_{0}/v$. Since the CS-based SAR imaging algorithm applied in the azimuth direction is essentially searching the most matched atom from a redundant dictionary Equation (15) to $s_{1}\left(t_{m}\right)$, the degree of match is mainly affected by the exponential term in Equation (54), whereas the minor shift of the azimuth windowing function has little influence. Assuming that the most matched atom in the redundant dictionary $\Phi_{1}$ is

(55) $$\begin{array}{c} s_{0}\left(t_{m}-k^{\prime }\Delta \tau \right)=\omega_{a}\left(t_{m}-k^{\prime }\Delta \tau \right)\exp\left(-j2\pi \frac{v^{2}}{\lambda R_{B}}{\left(t_{m}-k^{\prime }\Delta \tau \right)}^{2}\right). \end{array}$$

When $k^{\prime }\Delta \tau =k\Delta \tau +\frac{v_{r}R_{B}}{v^{2}}$, $s_{1}\left(t_{m}\right)$ matches the atom most, resulting in $k^{\prime }=k+\frac{v_{r}R_{B}}{v^{2}\Delta \tau }$. Obviously, for a stationary clutter with $v_{r}=0$ m/s, the reflection coefficient of the stationary clutter locates at the ${\left(N/2+k\right)}^{th}$ pixel in the azimuth direction. For a moving target with $v_{r}\neq 0$, the cross-track velocity of the moving target leads to an azimuth deviation of $\frac{v_{r}R_{B}}{v^{2}\Delta \tau }$ pixels, compared with that of the stationary clutter, on the SAR image reconstructed using the HVB-DCS algorithm.

**Figure 5:**
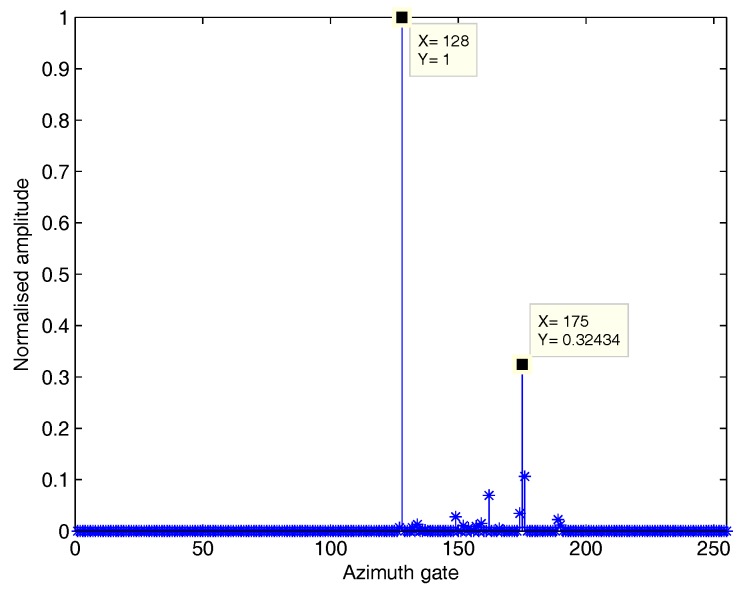
The influence of cross-track velocity on SAR image.

The influence of cross-track velocity is demonstrated in Figure 5. In Figure 5, a static scattering center and a moving scattering center with the cross-track velocity of $v_{r}=0.5$ m/s and the along-track velocity of $v_{a}=0$ m/s are both at (7071,0) m. The amplitude of reflection coefficient of the moving target is half that of the stationary clutter. The moving target has a deviation of 47 pixels in the azimuth direction compared with that of the stationary clutter on SAR image, which completely conforms to $\frac{v_{r}R_{B}}{v^{2}\Delta \tau }$.

#### 6.1.2. The Effect of Along-Track Velocity

For the analysis on along-track velocity, it is assumed that the moving target is at $\left(R_{B},0\right)$, with a given along-track velocity $v_{a}$ and cross-track velocity of $v_{r}=0$ m/s. Thus the received signal of the moving target, after demodulation and range compression, can be written as

(56) $$\begin{array}{c} \begin{array}{c} s_{1}\left(t_{m}\right)=\rho_{0}^{\left(1\right)}\omega_{a}\left(t_{m}\right)\exp\left(-j2\pi \frac{{\left(v-v_{a}\right)}^{2}}{\lambda R_{B}}t_{m}^{2}\right). \end{array} \end{array}$$

**Figure 6:**
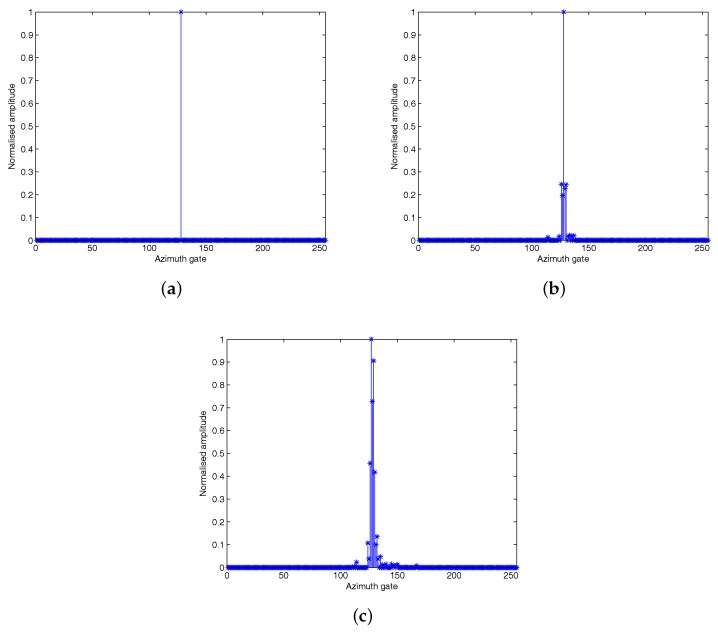
The influence of along-track velocity on SAR image: (**a**) $v_{a}=0$ m/s; (**b**) $v_{a}=1$ m/s; (**c**) $v_{a}=2$ m/s.

Similarly, for channel one, the received signal after range compression can be represented linearly by the atoms in the redundant dictionary $\Phi_{1}$, as

(57) $$\begin{array}{c} s_{1}\left(t_{m}\right)=\sum_{i=-N/2+1}^{N/2}C_{i}^{\left(1\right)}\omega_{a}\left(t_{m}-i\Delta \tau \right)\exp\left(-j2\pi \frac{v^{2}}{\lambda R_{B}}{\left(t_{m}-i\Delta \tau \right)}^{2}\right), \end{array}$$

where $C_{i}^{\left(1\right)}$ denotes the reconstructed reflection coefficient in channel one. Substituting Equation (56) into Equation (57), and dividing both sides by the item $\exp\left(-j2\pi \frac{v^{2}}{\lambda R_{B}}t_{m}^{2}\right)$, results in

(58) $$\begin{array}{c} \rho_{0}^{\left(1\right)}\omega_{a}\left(t_{m}\right)\exp\left(-j2\pi \frac{\left(v_{a}^{2}-2vv_{a}\right)}{\lambda R_{B}}t_{m}^{2}\right)=\sum_{i=-N/2+1}^{N/2}D_{i}^{\left(1\right)}\omega_{a}\left(t_{m}-i\Delta \tau \right)\exp\left(-ji\omega t_{m}\right), \end{array}$$

where $\omega =\frac{-4\pi v^{2}}{\lambda R_{B}}\Delta \tau$, and $D_{i}^{\left(1\right)}=C_{i}^{\left(1\right)}\exp\left(-j\frac{2\pi v^{2}}{\lambda R_{B}}{\left(i\Delta \tau \right)}^{2}\right)$. When the along-track velocity $v_{a}$ of the moving target equals zero, from Equation (58), we can see that the left side of the equation equals a constant. Thus on the right side of the equation, the most sparse solution is $D_{i}^{\left(1\right)}=0\;\left(i\neq 0\right)$ and $D_{0}^{\left(1\right)}=\rho_{0}^{\left(1\right)}$. In general, the along-track velocity of the target is much less than that of the flying platform, resulting in a slow variation of the terms on the left side of Equation (58). Thus the left side of Equation (58) contains only a few low frequency components. With the increase of left side of Equation (58), the high frequency component increases slowly. $D_{i}^{\left(1\right)}$ can be approximated to the coefficients of expansion of the slowly varying function under different frequency components. Therefore, with the increase of the left side of the Equation (58), the nonzero value $D_{i}^{\left(1\right)}$ will spread to both sides, centering at $D_{0}^{\left(1\right)}$, which leads to the defocus of the moving target on the SAR image. The influence of along-track velocity can be shown in Figure 6, where $v_{a}$ is set as different values of 0 m/s, 1 m/s and 2 m/s. It can be seen from Figure 6 that as the along-track velocity increases, the azimuth defocus of the moving target on the SAR image becomes more serious.

The defocus of the moving target on the SAR image can be treated as a basis mismatch problem [44]. In this work, the measurement matrices are built based on the echo signal models of two channels (Equations (13) and (19) in Section 4.1), assuming that the along-track velocity $v_{a}$ equals zero. However, in practice, this assumption does not always hold true. In this case, the constructed measurement matrix does not exactly match the underlying true one, leading to the basis mismatch problem. A parameter $\beta_{d}$ is defined in [44] to quantitatively evaluate the difference between the assumed basis and the true one. The larger the absolute value of $v_{a}$ is, the higher the mismatch level of the constructed measurement matrix will be, resulting in a higher $\beta_{d}$. Moreover, the defocus effect can be evaluated by the reconstruction error of the sparse vector recovered with mismatched basis. Based on [44], an upper bound of the $\ell_{1}$-norm reconstruction error is defined as:

(59) $${∥\rho_{k}-{\hat{\rho }}_{k}∥}_{1}\leq N\beta_{d}{∥\rho_{k}∥}_{q},$$

where $\rho_{k}$ and ${\hat{\rho }}_{k}$ denote the true and reconstructed sparse vector respectively, *N* is the dimension of the sparse vector, ${∥\cdot ∥}_{q}$ represents the $\ell_{q}$-norm, and 1≤q≤∞. Thus, the azimuth defocus on the SAR image is bounded given $v_{a}$. In addition, please refer to [45,46] for more analyses of basis mismatch problem in CS-based SAR imaging and the methods to deal with it.

Furthermore, the effect of $v_{a}$ on the azimuth defocus is analyzed through simulation. The degree of azimuth defocus is represented by the relative reconstruction error, which is defined as

(60) $$err≜{∥\rho_{k}-{\hat{\rho }}_{k}∥}_{1}/{∥\rho_{k}∥}_{1}.$$

**Figure 7:**
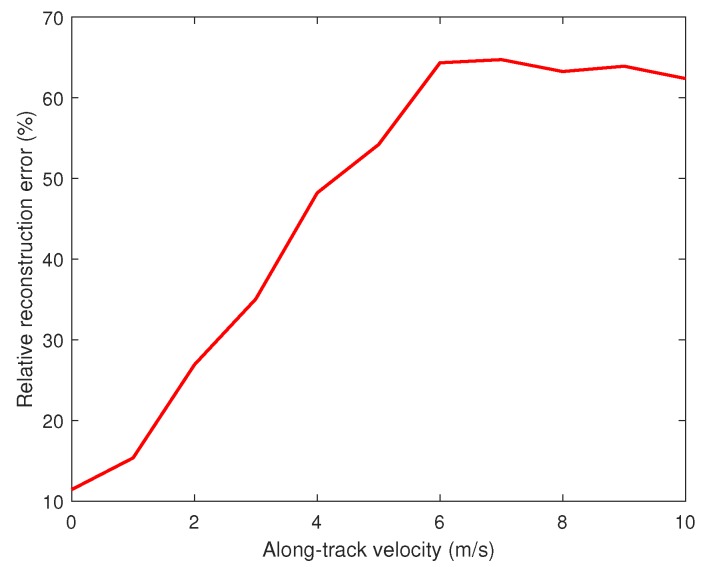
Relative reconstruction error versus along-track velocity.

Figure 7 shows the relative reconstruction error err versus $v_{a}$. The result illustrates that the reconstruction error err is low when the absolute value of $v_{a}$ is relatively small.

### 6.2. The Simulation on Point Targets

In this section, the performance of the HVB-DCS algorithm is verified by simulations on point targets. The nearest slant range $R_{B}$ is set as 7071 m. Four scattering centers are located at the range bin $R_{B}=7071$ m, including three static scattering centers, and a moving scattering center with cross-track velocity of $v_{r}=0.5$ m/s and along-track velocity of $v_{a}=0$ m/s. The three stationary targets are located at −5 m, 0 m, +5 m in the azimuth direction, and the moving target is located at 0 m in the azimuth direction. The amplitudes of reflection coefficient of three stationary targets are the same, which are twice that of the moving target. The parameters of the simulated SAR radar system are the same as those listed in Table 1.

**Figure 8:**
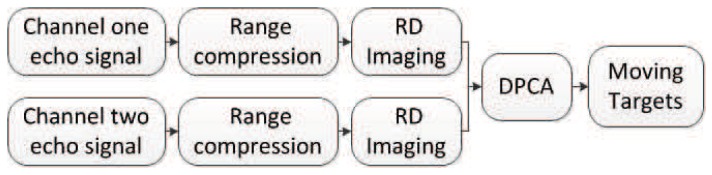
Flow chart of the RD based GMTI system.

**Figure 9:**
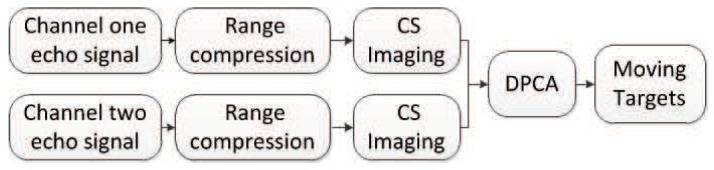
Flow chart of the CS-based GMTI system.

**Figure 10:**
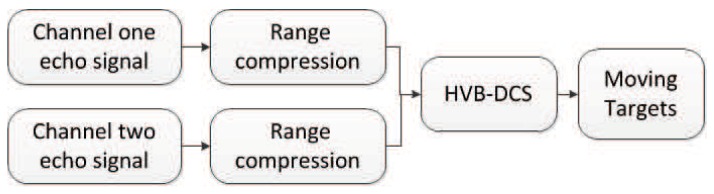
Flow chart of the HVB-DCS based GMTI system.

In the field of SAR-GMTI, the proposed HVB-DCS algorithm is compared to the RD [42] and CS [43] algorithms. The flow charts of the RD based GMTI system, the CS-based GMTI system, and the HVB-DCS based GMTI system are shown in Figure 8, Figure 9 and Figure 10, respectively. For the RD based GMTI system and CS-based GMTI system, an additional DPCA [47] procedure is needed for moving-target indication, which is shown in both Figure 8 and Figure 9. DPCA can effectively suppress clutter and detect slow moving targets. In contrast, in the HVB-DCS based GMTI system, the moving targets (the innovation components) and the stationary targets (the common component) can be directly distinguished using the DCS framework. Thus the DPCA is no longer required in the HVB-DCS based GMTI system and removed (Figure 10). This significantly simplifies the architecture of the GMTI system, and saves the computing time.

**Figure 11:**
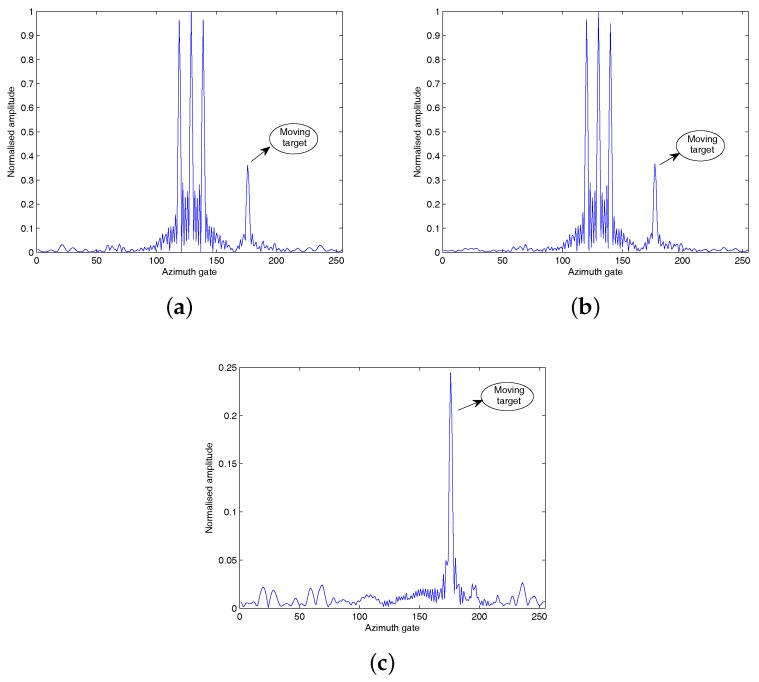
RD based reconstruction results: (**a**) channel 1 with oversampled raw data; (**b**) channel 2 with oversampled raw data; (**c**) DPCA with oversampled raw data.

**Figure 12:**
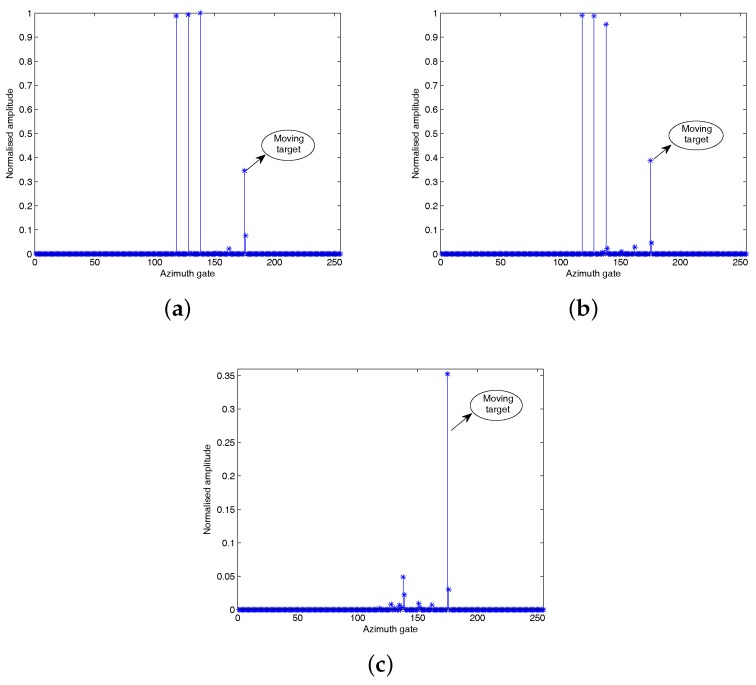
CS-based reconstruction results: (**a**) channel 1 with data rate 50% in azimuth; (**b**) channel 2 with data rate 50% in azimuth; (**c**) DPCA with data rate 50% in azimuth.

**Figure 13:**
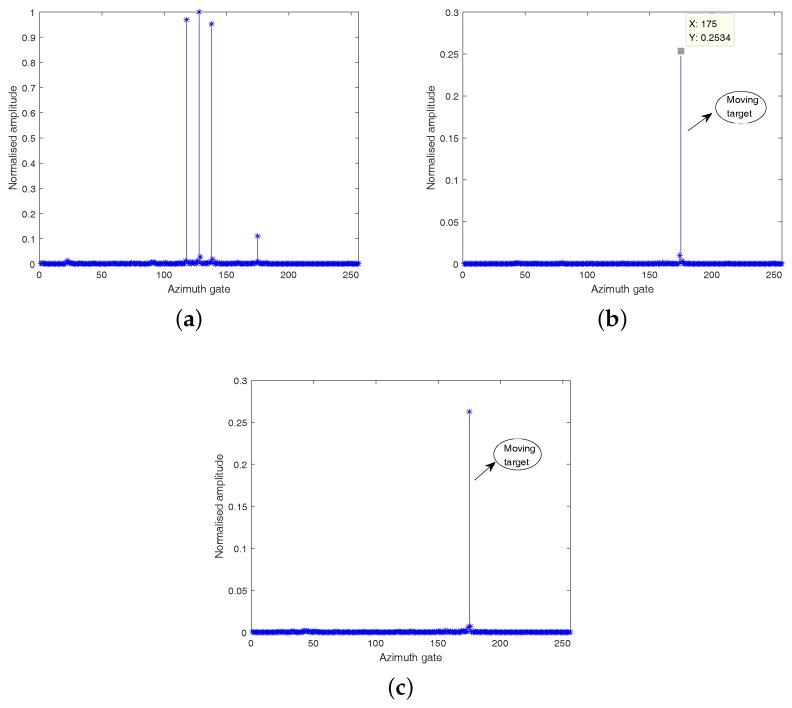
HVB-DCS based reconstruction results with data rate 37.5% in azimuth: (**a**) static scattering centers; (**b**) moving target in channel one; (**c**) moving target in channel two.

**Figure 14:**
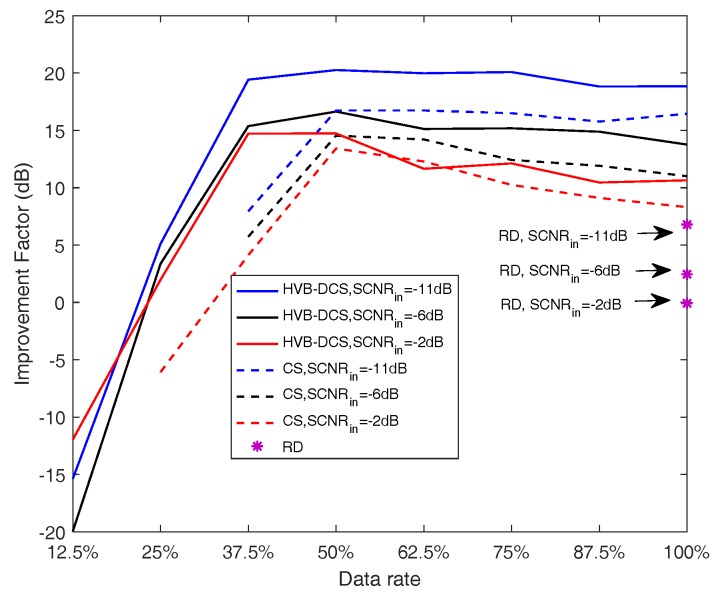
IFs of the RD, CS and HVB-DCS algorithms, based GMTI system for the varying data rates in azimuth and different ${\mathrm{SCNR}}_{\mathrm{in}}$ levels.

Figure 8, Figure 9 and Figure 10 show that all the three algorithms, i.e., RD, CS and HVB-DCS algorithms, can effectively identify the moving target. For the RD algorithm, we use the oversampled raw data to image. The result of the RD algorithm is shown in Figure 11. As a widely used SAR imaging algorithm, the RD algorithm has very high running speed, but the on-board memory required by the sampling rate is large. For the CS algorithm, the simulated radar system works in the random sampling mode introduced in Section 4.1. In our experiment, we choose randomly half of transmitted pulses in azimuth and recover the complex-valued SAR image based on the CS algorithm. The result is shown in Figure 12, which shows that the image reconstructed by using the CS algorithm is more clear, and the side-lobes are effectively suppressed. However, the computational cost of the CS algorithm is relatively high. For the HVB-DCS algorithm, we further drop randomly 62.5% of the transmitted pulses in azimuth to recover the SAR image. In our experiment, we reduce to about 37.5% of the original samples in the azimuth direction and reconstruct the SAR image using the HVB-DCS algorithm. The result is shown in Figure 13, which shows that the HVB-DCS algorithm has a great suppression effect on stationary clutter and is capable of identifying the moving target accurately. The effects of both the data rate in azimuth, and noise/clutter level on the detection performance of moving targets are tested by simulations. The adopted metric is the improvement factor (IF), which is defined as IF = ${\mathrm{SCNR}}_{\mathrm{out}}$/${\mathrm{SCNR}}_{\mathrm{in}}$, where ${\mathrm{SCNR}}_{\mathrm{in}}$ and ${\mathrm{SCNR}}_{\mathrm{out}}$ are the signal-to-clutter-noise ratios of input signal and output signal, respectively. We test the HVB-DCS and CS algorithms to detect moving targets by varying the data rate in the azimuth direction, at different ${\mathrm{SCNR}}_{\mathrm{in}}$ levels, except that the performance of RD algorithm is tested at different ${\mathrm{SCNR}}_{\mathrm{in}}$ levels with data rate 100%. The IFs averaged over 100 Monte Carlo trials are shown in Figure 14. For the HVB-DCS algorithm, at the same ${\mathrm{SCNR}}_{\mathrm{in}}$ level, the IF first increases dramatically with the data rate in azimuth. When the data rate in the azimuth direction exceeds 37.5%, the IF grows slowly. For the CS algorithm, at the clutter/noise levels of ${\mathrm{SCNR}}_{\mathrm{in}}=-11$ dB and ${\mathrm{SCNR}}_{\mathrm{in}}=-6$ dB, the IF is negative infinite when the data rate in azimuth is lower than 37.5%. Furthermore, at the clutter/noise level of ${\mathrm{SCNR}}_{\mathrm{in}}=-2$ dB, the IFs are negative infinite when the data rate in azimuth is lower than 25%. With the data rate in azimuth approaching 37.5%, the IF increases to a value larger than zero. When the data rate in the azimuth direction increases to more than 50%, the IF gradually increases with the data rate in azimuth at the same ${\mathrm{SCNR}}_{\mathrm{in}}$ level. The main reason is that when the data rate in azimuth decreases, the reconstruction error increases gradually, thus affecting the performance of clutter suppression. Moreover, it is noted that the IFs of HVB-DCS algorithm with the data rate 37.5% in azimuth are almost at the same level with those of CS algorithm with the data rate 50% in azimuth. The IFs of RD algorithm (indicated by asterisks) are the lowest among the three algorithms. This verifies that the proposed HVB-DCS algorithm outperforms the CS and RD algorithms in terms of clutter suppression and moving target detection performance.

**Figure 15:**
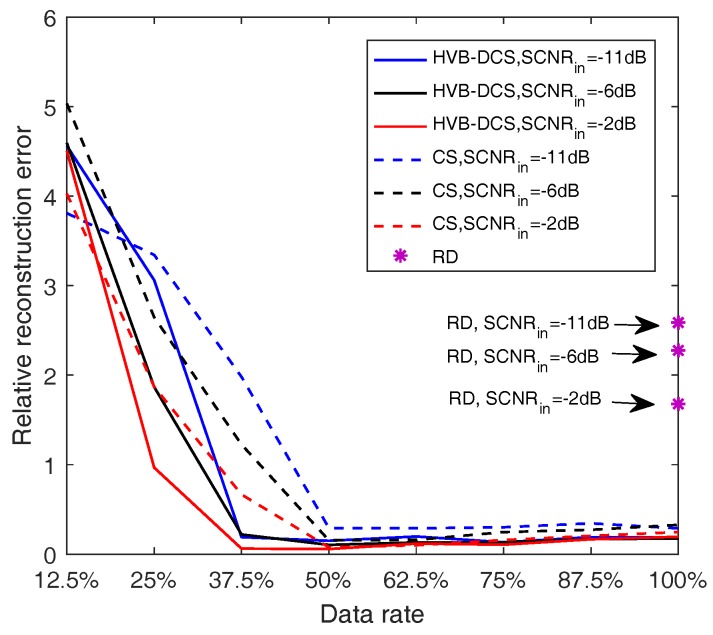
Reconstruction errors of three algorithms, i.e., RD, CS and HVB-DCS algorithms, based GMTI system for the varying data rates in azimuth and different ${\mathrm{SCNR}}_{\mathrm{in}}$ levels.

To examine the reconstruction accuracy of the proposed HVB-DCS algorithm, we introduce the reconstruction error, which is defined as

(61) $$e_{rec}=\frac{\sum_{l=1}^{L}{∥{\hat{x}}_{l}-x_{l}∥}_{2}}{\sum_{l=1}^{L}{∥x_{l}∥}_{2}},$$

where *L* is the number of range bins, $x_{l}$ and ${\hat{x}}_{l}$ denote the true and reconstructed sparse vector at the $l^{th}$ range bin, respectively. The reconstruction errors of the RD, CS and HVB-DCS algorithms, are calculated by varying data rates in azimuth, at different ${\mathrm{SCNR}}_{\mathrm{in}}$ levels. The results are shown in Figure 15. The reconstruction error of the HVB-DCS algorithm is lower than 0.2 when the data rate is not less than 37.5%, whereas the reconstruction error of the CS algorithm is lower than 0.3 when the data rate is not less than 50%. In addition, compared to the CS algorithm, the HVB-DCS algorithm has smaller reconstruction errors when the data rate exceeds 37.5%. The main reasons are due to the following two aspects. First, the DCS exploits both intra-signal and inter-signal correlations, which ensures a good correlation between the channels in the case of lower data rate. Secondly, the hierarchical structure is more suitable for complex models. Moreover, the higher the noise/clutter level is, the larger the reconstruction errors are, which shows that the detection performance degrades with the increase of the noise/clutter level.

### 6.3. Effects of Problem Size and Data Rate on Complexity of HVB-DCS

**Figure 16:**
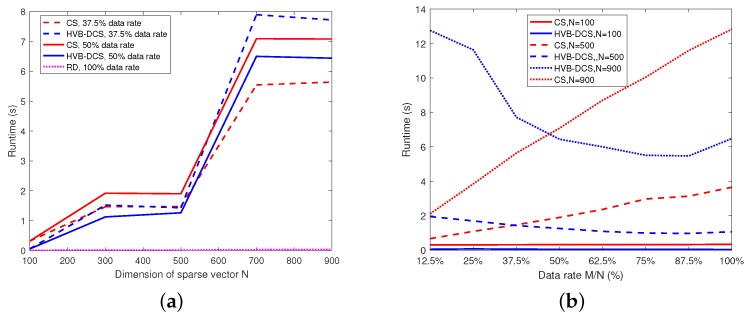
Complexity comparisons of the RD, CS and HVB-DCS algorithms: (**a**) runtime versus sparse vector dimension *N*; (**b**) runtime versus data rate M/N.

The goal of this section is to analyze the computational complexity of the HVB-DCS algorithm in terms of problem size and data rate using simulations. The computational complexity of the HVB-DCS algorithm is further compared with those of the RD and CS-based algorithms. First, we set the data rate as 37.5% and 50% for the HVB-DCS and CS-based algorithms respectively, and 100% for the RD algorithm. Meanwhile, we vary the dimension of sparse vector *N* (also, the number of samples in the azimuth direction) in the range of [100,900]. We plot the runtime (averaged over 100 trials) of the HVB-DCS, RD and CS-based algorithms versus *N* in Figure 16a. The results show that the RD algorithm achieves the shortest runtime. When the data rate is at the level of 37.5% and N≤500, the proposed HVB-DCS algorithm takes almost the same runtime with the CS-based algorithm to perform moving target indication. When the data rate is at the level of 37.5% and N>500, the proposed HVB-DCS algorithm takes longer runtime than the CS-based algorithm. When the data rate is at the level of 50%, the runtime of HVB-DCS is shorter than that of the CS-based algorithm in the range of [100,900].

In addition, we examine the runtime of the HVB-DCS and CS-based algorithms versus data rate when setting *N* at different levels, i.e., 100, 500 and 900 respectively. The data rate varies in the range of [12.5%,100%] for the two algorithms. The results are shown in Figure 16b. When N=100, the runtime of HVB-DCS is shorter than that of the CS-based algorithm at any data rate in the range of [12.5%,100%]. When N=500 and the data rate is larger than 37.5%, HVB-DCS takes shorter runtime than that of the CS-based algorithm, and the similar result occurs when N=900 and the data rate is larger than 50%.

### 6.4. Experiment on Real SAR Data

In order to verify the performance of the proposed HVB-DCS algorithm in practice, we carry out an experiment on data from a real background cluttered environment, which are collected by the satellite RADARSAT-1 in the Vancouver region [42]. The parameters of RADARSAT-1 satellite are shown in Table 2.

In the experiment, the real SAR data from single-channel SAR system (RADARSAT-1) is used to simulate dual-channel raw data. The detailed procedures are as follows. According to the imaging geometry models for point targets as in Equations (13) and (19) in Section 4.1, there is a phase difference $\exp\left(-j\pi d^{2}/\left(2\lambda R_{B}\right)\right)$ and a time delay $d/\left(2\left(v-v_{a}\right)\right)$ between the received signals from two channels. First, all the real data from the first channel is delayed Δt time, and then multiplied with $\exp\left(-j\pi d^{2}/\left(2\lambda R_{B}\right)\right)$, to produce the raw data from the second channel of the simulated dual-channel SAR system. Secondly, in order to simulate the channel imbalance, some random amplitudes and phase errors are added to the raw data of the second channel. Finally, the echo signals of nine targets (including five stationary targets and four moving targets moving) are added to the raw data of both channels. Based on the raw data from the simulated dual-channel SAR system, three algorithms, e.g., the RD, CS and HVB-DCS algorithms, are utilized to detect the moving targets.

**Figure 17:**
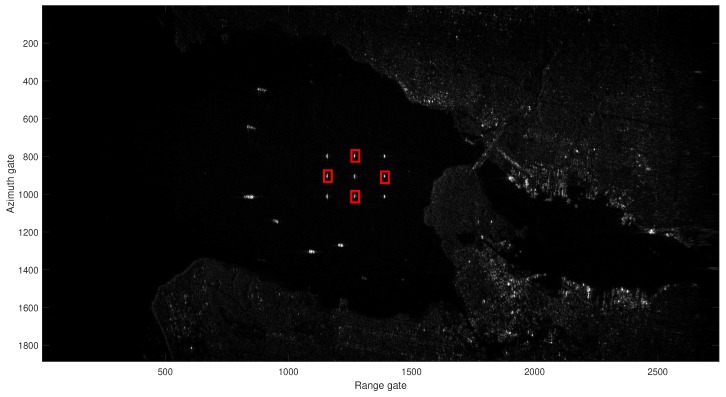
Ground truth scene containing the static clutter, five stationary targets and four targets with same cross-track velocity of $v_{r}=5$ m/s, and same along-track velocity of $v_{a}=5$ m/s. The red rectangle indicates the moving target.

Figure 17 shows the SAR image of the observation scene, which contains the static clutter, five stationary point targets and, four moving targets with the same cross-track velocity of $v_{r}=5$ m/s, and the same along-track velocity of $v_{a}=5$ m/s. The radar image covers an area of about 2.8 km in azimuth and 1.9 km in range. The corresponding parameters of nine targets are listed in Table 3. The four moving targets in full SAR RADARSAT-1 image are marked with the red rectangles in Figure 17. We use the oversampled raw data in azimuth for the RD algorithm, and 50% of the oversampled raw data in azimuth for CS algorithm and 37.5% of the oversampled raw data in azimuth for the HVB-DCS algorithm.

**Figure 18:**
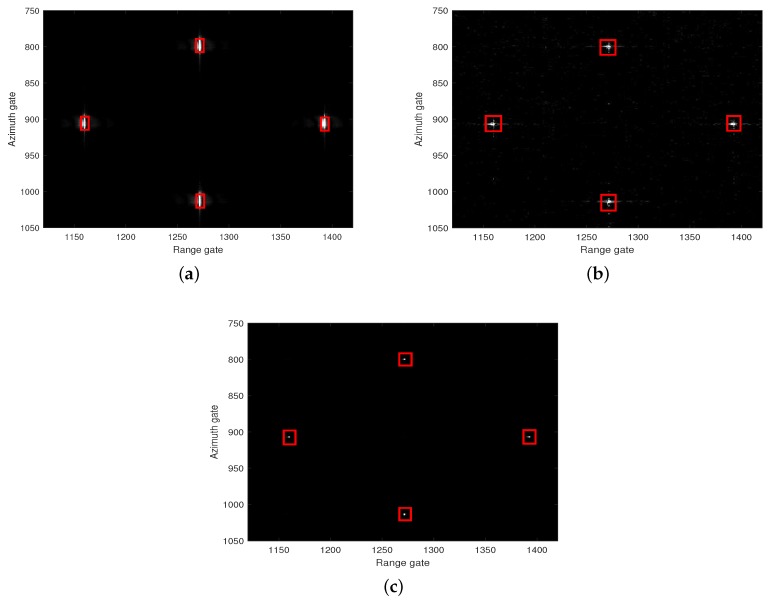
GMTI results: (**a**) RD with oversampled raw data; (**b**) CS with 50% of the oversampled raw data; (**c**) HVB-DCS with 37.5% of the oversampled raw data. The red rectangle indicates the moving target. The partial enlarged result is shown in the upper right corner of the SAR image.

GMTI results (Figure 18) show that all of the three algorithms can distinguish the moving targets from the stationary clutter successfully. Moreover, the final ${\mathrm{SCNR}}_{\mathrm{out}}$ of the three algorithms are calculated to evaluate detection performance. The initial ${\mathrm{SCNR}}_{\mathrm{in}}$ equals −69 dB. The ${\mathrm{SCNR}}_{\mathrm{out}}s$ of the SAR image recovered based on the RD, CS and HVB-DCS algorithms are −13 dB, −12 dB and −5 dB, respectively. Note that, there is an obvious increase in the SCNR. The RD algorithm has the lowest ${\mathrm{SCNR}}_{\mathrm{out}}$. The SAR image of CS-based algorithm has a much lower ${\mathrm{SCNR}}_{\mathrm{out}}$ (−12 dB) compared with the proposed HVB-DCS algorithm (−5 dB). This is due to the reason that in the CS-based GMTI system, the mismatch between channels leads to the failure in energy cancelation of static targets and clutters, whereas the proposed HVB-DCS algorithm takes advantage of the correlation between two channels and suppresses the clutter and noise efficiently, even at a much lower data rate in the azimuth direction.

## 7. Conclusions

In this paper, we propose a novel HVB-DCS algorithm for GMTI using the dual-channel SAR system. The results from both the simulation on point targets and the experiment on data from the real background cluttered environment show that the proposed HVB-DCS algorithm can successfully detect multiple moving targets, while suppressing the clutter efficiently. The proposed algorithm achieves better detection performance for the GMTI system, in the metric of IF and reconstruction error, compared with the RD and CS algorithms.

## Figures and Tables

**Table 1:** SAR radar system parameters for simulation.

Parameter	Value
Wavelength (m)	0.03
Range bandwidth (MHz)	150
Platform height (m)	5000
Platform velocity (m/s)	150
Incidence angle (${}^{\circ }$)	45
PRF (Hz)	300
Aperture size (m)	2
Channel distance (m)	1

**Table 2:** RADARSAT-1 parameters.

Parameter	Value
Slant range of scene center (km)	988.65
Beam squint angle (rad)	0.0279
Effective radar velocity (m/s)	7062
PRF (Hz)	1256.98
Sampling rate (MHz)	32.317
Range FM rate (MHz/μs)	0.72135
Pulse duration (μs)	41.75
Radar center frequency (MHz)	5300

**Table 3:** Simulation parameters of five stationary targets and four moving targets.

No.	Azimuth Coordinate (m)	Nearest Slant Range (km)	Along-Track Velocity $v_{a}$ (m/s)	Across-Track Velocity $v_{r}$ (m/s)
1	−500	0.8	5	5
2	−400	987.65	0	0
3	800	987.65	0	0
4	200	988.17	0	0
5	−1100	988.17	5	5
6	100	988.17	5	5
7	−500	988.73	5	5
8	−400	988.73	0	0
9	800	988.73	0	0
